# An Italian Survey on the Management of Pediatric Convulsive Status Epilepticus: More Than Just a Pharmacological Choice

**DOI:** 10.1002/brb3.70433

**Published:** 2025-03-31

**Authors:** Caterina Zanus, Giulia Cannizzaro, Giacomo Danieli, Angela Amigoni, Silvia Buratti, Francesca Izzo, Massimo Mastrangelo, Maria Cristina Mondardini, Paola Costa, Anna Rosati, Carla Marini, Lucia Fusco, Italian Paediatric Status Epilepticus Group

**Affiliations:** ^1^ Institute for Maternal and Child Health ‐ IRCCS “Burlo Garofolo” ‐ Trieste Italy; ^2^ Paediatric Neurology Unit, Meyer Children's Hospital IRCCS University of Florence Florence Italy; ^3^ Unit of Biostatistics, Epidemiology and Public Health, Department of Cardio‐Thoraco‐Vascular Sciences and Public Health University of Padua Padua Italy; ^4^ Paediatric Intensive Care Unit, Department of Woman's and Child's Health University Hospital of Padova Padua Italy; ^5^ Neonatal and Paediatric Intensive Care Unit, Emergency Department IRCCS Istituto Giannina Gaslini Genoa Italy; ^6^ Paediatric Intensive Care Unit Buzzi Children's Hospital Milan Italy; ^7^ UOC Terapia Intensiva Pediatrica Pre Post Chirurgica IRCCS Policlinico San Donato San Donato Milanese Italy; ^8^ Paediatric Anaesthesia and Intensive Care Unit IRCCS AOUBO Bologna Italy; ^9^ Child Neuropsychiatry, Department of Paediatrics Santa Chiara Hospital, Azienda Provinciale per i Servizi Sanitari Trento Italy; ^10^ Child Neurology and Psychiatric Unit, Paediatrics Hospital G. Salesi Azienda Ospedaliero Universitaria delle Marche Ancona Italy; ^11^ Department of Neuroscience Bambino Gesù Children's Hospital, IRCCS, Full Member of European Reference Network EPICARE Rome Italy

**Keywords:** intensive care unit, pediatric, status epilepticus, survey, treatment

## Abstract

**Background and Purpose:**

To explore specialists’ opinions on the current management of pediatric convulsive status epilepticus (CSE) in Italy and the main factors influencing the applicability of guidelines.

**Methods:**

We conducted a national survey of child neurologists, pediatric emergency physicians, and intensivists. Within the multidisciplinary Italian Paediatric Status Epilepticus (IPSE) Group, a web‐based 48‐multiple‐choice questionnaire was developed to explore treatment choices, use of internal protocols and guidelines, and self‐perceived competencies in the treatment of CSE.

**Results:**

Responses were received from 250 clinicians from 34 Italian hospitals (response rate 71%). Intravenous midazolam (iv‐MDZ) was the preferred benzodiazepine (BDZ) when iv access was available (90%). When iv‐access was unavailable, 75% of clinicians used BDZs; rectal diazepam was the most indicated (65.6%). Concerning second‐line treatment, the choices were equally distributed between phenytoin (55.2%), levetiracetam (52.4%), and phenobarbital (52.4%). MDZ infusion at a dosage < 0.23 mg/kg/h was also a frequent choice (38%). A PICU in the hospital influenced this latter choice, resulting in a significantly greater use of iv‐MDZ by pediatric emergency physicians working in these hospitals. Answers’ variability was related to organizational aspects such as the availability of on‐duty specialists and diagnostic tools in emergency settings.

**Conclusions:**

This survey confirmed that first‐line treatment of pediatric CSE relied on iv‐MDZ and that the heterogeneity of therapeutic choices started from the second‐line treatment in real life. The survey also highlighted the need to consider the organizational heterogeneity among settings and to involve different specialties in an integrated and feasible approach.

## Graphical abstract

A survey exploring specialists’ opinions on the current management of pediatric convulsive status epilepticus (CSE) in Italy confirms that first‐line treatment of pediatric CSE relies on intravenous MDZ and that heterogeneity of therapeutic choices starts from the second‐line treatment. Answers’ variability concerning organizational aspects such as the availability of on‐duty specialists and diagnostic tools in emergency settings highlights the need to consider the organizational heterogeneity among settings and to involve different specialties for an integrated and feasible approach.

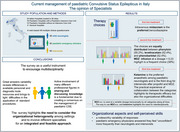

## 1 Introduction

Status epilepticus (SE) is one of the most common neurologic emergencies in children, with an overall mortality of 3.6% (Neligan et al. 2019). Recently, a trend toward lower mortality has occurred, but morbidity, including neuro‐disability, learning difficulties, and new‐onset epilepsy, may reach up to 22% (Messahel et al. 2022). The high percentage of neurological sequelae raises several questions concerning treatment‐related aspects of care and adherence to guidelines developed so far.

Management of convulsive status epilepticus (CSE) is a time‐sensitive medical emergency due to the necessity to cope with rapid biochemical and electrophysiological brain modifications that cause concomitant changes in the response to antiseizure medications (ASMs) and make seizure cessation less likely (Naylor 2023). Current CSE treatment guidelines consist of a timed‐sequential administration of anticonvulsant drugs, starting with benzodiazepines (BZDs, first‐line treatment), switching to non‐BZD ASMs (second‐line treatment), and proceeding with anesthetics (third‐line treatment) in intensive care units (ICUs) (Brophy et al. 2012; Glauser et al. 2016). Management of CSE requires a multidisciplinary team, including pediatric emergency physicians, child neurologists, and intensivists, working together to deliver comprehensive care as outlined in the current guidelines.

The literature indicates that healthcare providers have not broadly and coherently adhered to the current SE guidelines. Deviations from the guidelines in the treatment of SE are frequent, ranging from 10.7% to 66.1%; the principal causes include the difficulty in defining the exact time of seizure duration, delay in the decision to administer ASMs, different experiences of the professionals involved, gaps in knowledge, and problems with drug availability (Hill et al. 2017).

Moreover, although the first‐line therapy is the administration of BDZ in all guidelines, the choice of drugs for second‐ or third‐line treatment and the timing of the general anesthetic drugs for RSE are challenging in pediatric patients (Ulusoy et al. 2019). Still, it demands careful interpretation and application of evidence (Appleton 2020). In particular, the total number of second‐line ASMs, the time to start midazolam (MDZ) infusion, and its dosage can vary.

The few published surveys on pediatric SE management show heterogeneity in the approach that increases after the first‐line treatment, becoming particularly evident in the later stages, which requires the involvement of both epileptologists and intensivists (Nuñez et al. 2023; Dedeoglu et al. 2023; Suthar et al. 2024).

The Italian Paediatric Status Epilepticus (IPSE) Group is a multidisciplinary working group that includes child neurologists, pediatricians, and intensivists. It was founded in June 2022 as a non‐profit organization to improve pediatric CSE diagnosis, treatment, and management in Italy.

The initial task force included specialists working on the 11 trial sites of the KETASER01 study, a non‐profit randomized study on the effectiveness of ketamine (KE) in CSE in pediatric age (Study KETASER01‐ EudraCT Number: 2013‐004396‐12). The study required adherence to a protocol with a standardized second‐line treatment, which included phenytoin, phenobarbital, and MDZ infusion before the randomization to a third‐line anesthetic (Rosati et al. 2016). Specialists involved were child neurologists, pediatric emergency physicians, and intensivists. The subsequent critical analysis of the study identified the necessity to respect a standardized therapeutic protocol for inclusion and the involvement of many different participating actors among the principal factors causing insufficient recruitment and the premature closure of the study (Rosati et al. 2022).

The contribution of experience and knowledge of each actor potentially involved in the feasible and pragmatic development of treatment protocols was an essential clue to improving adherence (Limotai et al. 2020).

One of the IPSE Group's first steps was, therefore, to create a survey with the aim of obtaining active stakeholder involvement in sharing information about current practices at individual institutions and starting to explore the main barriers to the applicability of guidelines and protocols on the management of CSE.

## 2 Methods

From January to March 2023, the IPSE group held targeted meetings to develop a questionnaire based on current guidelines and principal articles on pediatric SE management. The survey consisted of 48 multiple‐choice questions. The following categories were covered: specialty and hospital affiliation of participants; level of care intensity and operational setting of the hospital; availability of on‐duty specialists; availability of EEG and diagnostic tools; choice of first‐, second‐, and third‐line medications; use of internal protocols and guidelines; level of self‐confidence in recognizing and managing patients with CSE. No patient data were requested. The questionnaire was sent via Google Forms and emailed to all member specialists of the IPSE Group with a cover letter explaining the study's rationale and a request to inform and involve other colleagues in the survey. Responses were received between March and July 2023. The questionnaire is available as online supporting information.

### 2.1 Participants

Starting from the neurologists and intensivists of the 11 tertiary centers who participated in the KETASER01 study, the survey then involved clinicians of 34 Italian hospitals located in 30 cities and 17 regions and with different characteristics and quality of care: 6 were general hospitals, the others were pediatric hospitals, among which 16 had a pediatric ICU.

Participants were physicians from emergency rooms, pediatrics and children's neurology departments, and ICUs, representing all specialties involved in the multidisciplinary management of childhood SE. The recruitment was committed to the members belonging to the initial IPSE network, regardless of their role (ER pediatrician, neurologist, or intensivist), with the task of engaging colleagues involved in the treatment of pediatric CSE.

### 2.2 Statistical Analysis

Data were managed and analyzed with R statistical software (R version 4.2.3, 2023‐03‐15 ucrt). We utilized descriptive statistics to clarify questionnaire responses. The survey data were exclusively categorical, and we employed the Chi‐square (*χ* ^2^) tests to assess the associations and dependencies between these categorical variables. We considered *p* values of ≤ 0.05 as statistically significant. Some questions allowed for the possibility of providing more than one answer. In some cases, all combinations of responses were selected, grouping them appropriately; in others, the responses were analyzed individually about a specific drug, creating a dichotomous variable that encoded whether there was a response to the drug under analysis. Specifically, we applied this method to analyze the choice of levetiracetam (LEV) and intravenous MDZ (iv‐MDZ) as a second‐line drug and the option of KE as a third‐line drug. Due to the variability in the number of participants of each hospital and the heterogeneous organizational characteristics, we evaluated the presence or absence of an exclusively pediatric ICU as the only organizational grouping criterion, distinguishing two categories of hospitals. We considered only hospitals with a minimum of five patients to analyze participants' level of awareness on the use of regional references, care pathway (CP), or guidelines.

## 3 Results

### 3.1 Participation

We invited 352 clinicians to participate and receive the web survey link. Responses were obtained from 250 clinicians (71%), homogenously covering the three specializations: 82 were pediatric emergency physicians, 88 were child neurologists, and 80 were intensivists (Table 1).

**TABLE 1:** Participant's specialization and hospital level of care.

Physician specialization	Number (*N* = 250)	Percentage
**Emergency pediatrician**	**82**	**32.8%**
Working in a hospital with an exclusively pediatric ICU	45	55%
Working in a hospital with a non‐exclusively pediatric ICU	37	45%
**Pediatric neurologist**	**88**	**35.2%**
Working in a hospital with an exclusively pediatric ICU	48	54.5%
Working in a hospital with a non‐exclusively pediatric ICU	40	45.5 %
**Intensivist**	**80**	**32%**
Working in a hospital with an exclusively pediatric ICU	48	60%
Working in a hospital with a non‐exclusively pediatric ICU	32	40%

Abbreviation: ICU, intensive care unit.

### 3.2 Therapy Choices

**FIGURE 1:**
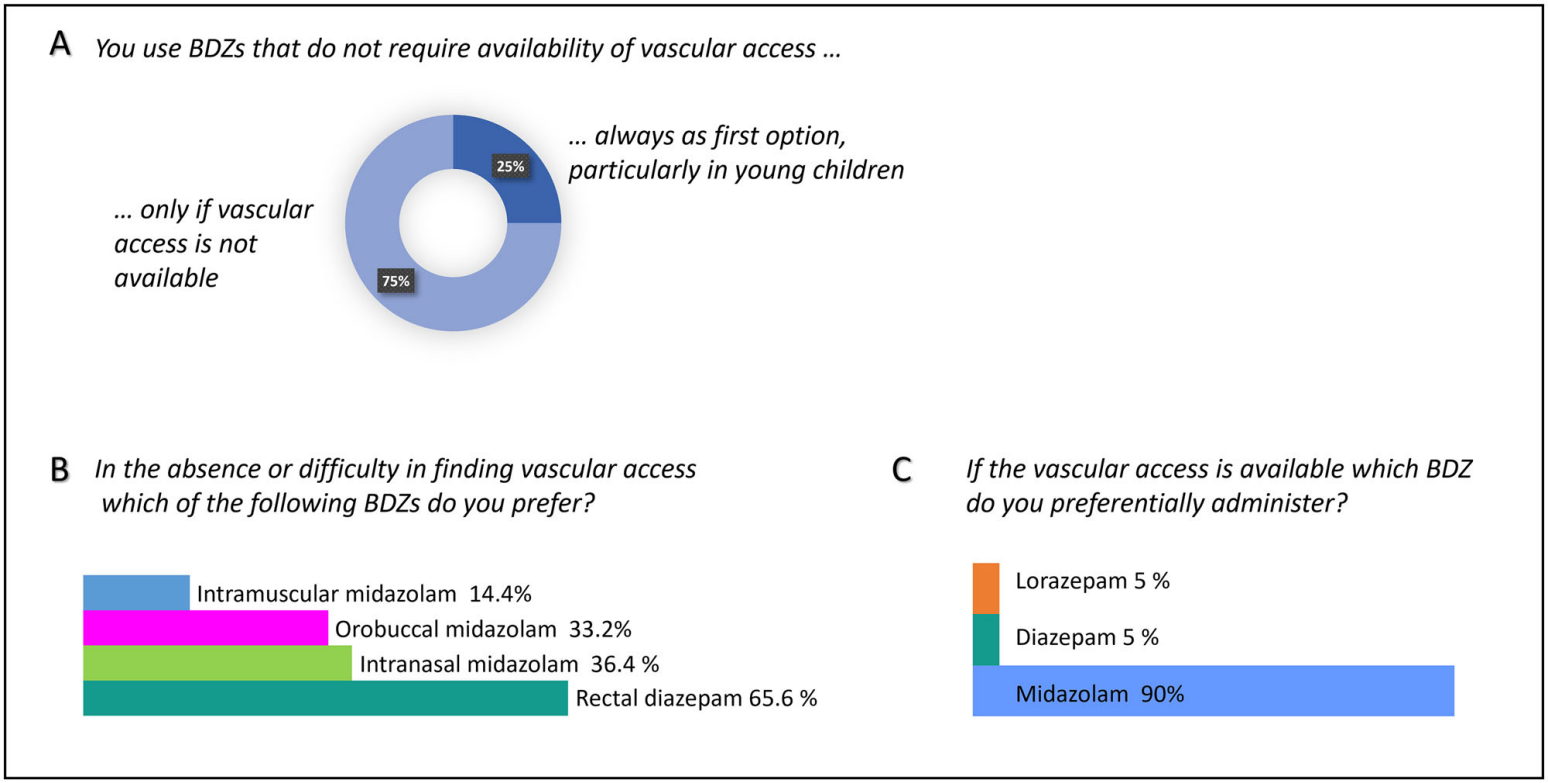
First‐line therapy choices. (A) Participants’ answers to the survey question “You use BDZ that do not require availability of vascular access” (a single choice was possible between “only if vascular access is not available” and “always as first option, particularly in young children”). (B) Participants’ answers to the survey question “In the absence or difficulty in finding vascular access which of the following BDZs do you prefer?” (C) Participants’ answers to the survey question “If the vascular access is available which BDZ do you preferentially administer?” BDZ = benzodiazepines.

iv‐MDZ, used by 90% of the participants at a dosage < 0.2 mg/kg, was the preferred first‐line treatment in children presenting with SE, while diazepam (DPZ, 5%) and lorazepam (LZP, 5%) were rarely used. The preferred non‐iv BDZs were rectal DZP (65.6%), intranasal MDZ (36.4%), and buccal MDZ (33.2%); intramuscular MDZ was seldom administered (14.4%). 75% of participants chose the non‐iv route “only if vascular access was not available,” whereas 25% of physicians undertook the non‐iv route regardless of the presence or absence of iv‐access, especially in young children (Figure 1). Most participants (79.6%) declared to use a maximum of two doses of iv‐MDZ (0.1–0.2 mg/kg) before switching to a second‐line treatment.

**FIGURE 2:**
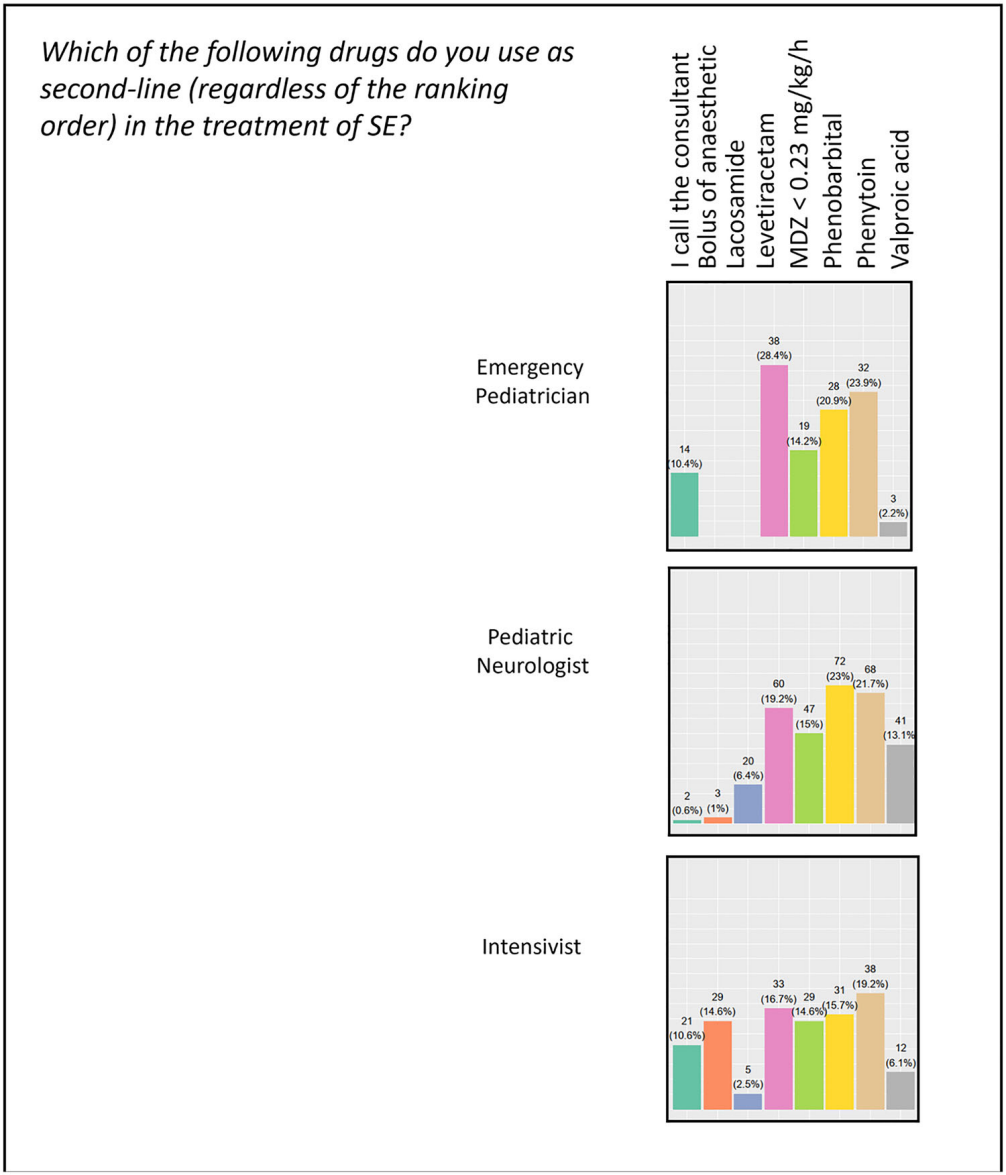
Second‐line therapy choices. Participants’ answers are divided by specialty (emergency pediatrician, pediatric neurologist, and intensivist) and second‐line treatment choices “Which of the following drugs do you use as second line, in the treatment of SE, regardless of the ranking order?” are reported. SE = status epilepticus.

Concerning second‐line therapy, the most frequently considered choices were phenytoin (PHT, 55.2%), LEV (52.4%), and phenobarbital (PB, 52.4%). The fourth drug considered was iv‐MDZ (16%). 38% of participants used an infusion of MDZ at a dosage < 0.23 mg/kg/h in association with other ASMs or alone (6.4%) at this step of the therapeutic pathway (Figure 2).

**FIGURE 3:**
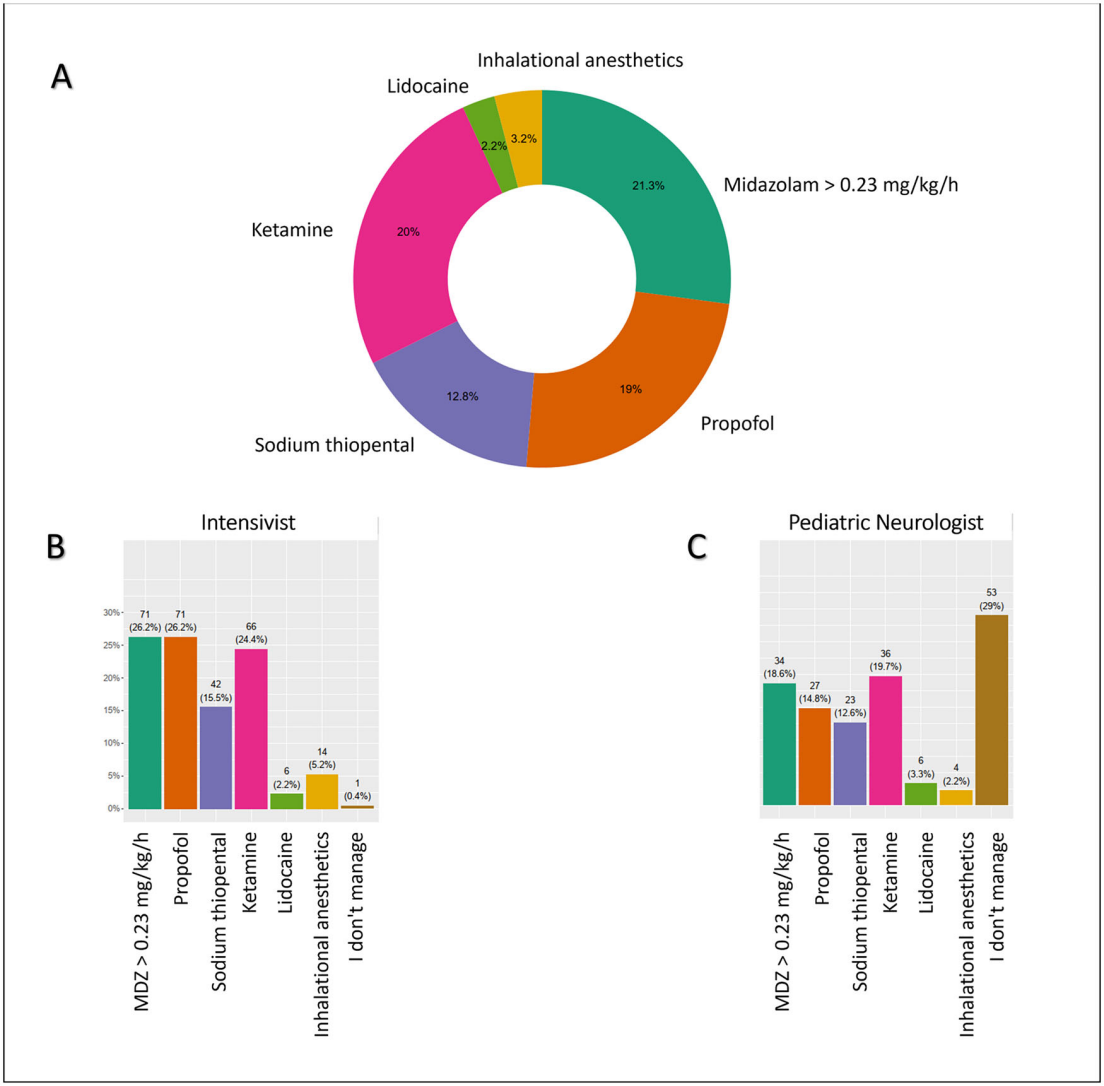
Third‐line therapy choices. Participants’ answers regarding third‐line treatment choices. (A) Overall drug choices are represented. (B and C) Drug choices of specialists are represented (respectively intensivist and pediatric neurologist).

Most child neurologists (65.9%), 55% of intensivists, and 15.9% of emergency pediatricians stated that they should administer at least two ASMs before considering an anesthetic drug. More than half (59.8%) of pediatric emergency physicians referred clinical decisions to child neurologists and/or intensivists. Figure 3 illustrates the wide distribution of choices concerning third‐line treatment for each specialty category.

### 3.3 Specialists Managing Therapy Choice

The following differences emerged from the analysis of the answers according to treating specialists: intensive care physicians use non‐iv BDZs only if iv‐access is unavailable (91% vs. 64%, *p* < 0.001). iv‐MDZ at dosage < 0.23 mg/kg/h as second‐line treatment was more frequently considered by child neurologists than pediatric emergency physicians (53% vs. 23%, *p* < 0.001). Child neurologists more frequently applied LEV than intensivists (68% vs. 41%, *p *< 0.001).

The majority (59.8%) of pediatric emergency physicians, 23.8% of intensivists, and 3.4% of child neurologists claimed that “it was not their responsibility to decide” when asked about the timing of the transition to third‐line therapy.

### 3.4 Factors Affecting the Care Level

A total of 22 out of the 34 participating hospitals could activate a team consisting of the neurologist and the neurophysiopathology technician, either present or on call, and perform an EEG in an emergency. Otherwise, child neurologists and technicians were on duty in only two hospitals 24 h daily.

A total of 16 hospitals had an exclusively pediatric ICU. In total, 24 (9.6%) participants from six general or pediatric hospitals started to transfer children with refractory SE (RSE) or super‐refractory SE (SRSE) to higher levels of specialized care hospitals.

We found significant differences in second‐line choices between hospitals with and without an exclusively pediatric ICU. Emergency pediatricians working in hospitals with an exclusively pediatric ICU, when asked which drugs they used as second‐line therapy (24% vs. 8.1%, *p* = 0.033), more frequently responded that they called a consultant and were more prone to use MDZ in infusion at a dosage < 0.23 mg/kg/h (24% vs. 14%) (Table 2).

**TABLE 2:** Second‐line therapy choices and level of care.

	All (*N* = 250)	Level 1	Level 2	*p* value
**Emergency pediatrician**				**0.033**
I call the consultant	14 (17%)	11 (24%)	3 (8.1%)	
Midazolam in infusion at dosage < 0.23 mg/kg/h	16 (20%)	11 (24%)	5 (14%)	
Other drugs administered as second‐line	52 (63%)	23 (51%)	29 (78%)	
**Pediatric neurologist**				0.5
I call the consultant	2 (2.3%)	1 (2.1%)	1 (2.5%)	
Midazolam in infusion at a dosage < 0.23 mg/kg/h	47 (53%)	23 (48%)	24 (60%)	
Other drugs administered as second‐line	39 (44%)	24 (50%)	15 (38%)	
**Intensivist**				0.14
I call the consultant	21 (26%)	13 (27%)	8 (25%)	
Midazolam in infusion at dosage < 0.23 mg/kg/h	23 (29%)	10 (21%)	13 (41%)	
Other drugs administered as second‐line	36 (45%)	25 (52%)	11 (34%)	

*Note*: Therapy choices are grouped into three categories, with particular attention to the use of midazolam in infusion at a dosage < 0.23 mg/kg/h; the category “other drugs administered as second‐line” includes also anesthetic drugs administered in bolus.

Abbreviations: ICU, intensive care unit; Level 1, hospital with an exclusively pediatric ICU; Level 2, hospital with a non‐exclusively pediatric ICU.

The level of care did not influence the number of drugs administered before the third‐line treatments.

### 3.5 Participants' Awareness of Organizational Aspects of Their Institution and Self‐Perceived Skills in Treatment

Questions related to organizational aspects resulted in a noteworthy variability of responses from the participants. Only four out of the 18 centers with at least five participants achieved 100% agreement on the question, “Do clinicians of your hospital use regional reference, CP or guidelines for the diagnosis and pharmacological treatment of SE?”

At the question “How confident do you feel about the treatment of status epilepticus?,” the most frequent answer was “rather confident.” Pediatric emergency physicians said they feel “unconfident” more frequently than neurologists and intensivists (25.6% vs. 4.5% and 10.1%, respectively).

## 4 Discussion

This study reported a national survey on pediatric CSE management involving emergency pediatricians, child neurologists, and intensivists. The overall number of responses expressed good participation, indicating widespread interest, involvement, and representativeness of all stakeholders in the different stages of SE management.

The survey confirmed that MDZ was the most widely used first‐line drug administered intravenously, while rectal DZP was the non‐iv BDZ most commonly used by pediatric emergency physicians. These results were consistent with other reports (Nuñez et al. 2023; Babl et al. 2009; Kaputu‐Kalala‐Malu et al. 2014; Wallace et al. 2019) and likely reflected multiple factors, including the evidence showing an overall benefit of BDZ habit, self‐confidence in one's personal experience, the age of the patient, and the availability of the drug (Zhao et al. 2016; McTague et al. 2018).

Dosage and number of BDZ doses were critical issues: the American Epilepsy Society recommended administering an adequate, single, full dose of BZD that could be repeated if seizures persisted (Glauser et al. 2016). However, a pattern of administering multiple smaller doses than recommended was commonly observed in real‐world practice (Sathe et al. 2019; Vasquez et al. 2020; Sathe et al. 2021; Noe 2021; Sheehan et al. 2021). Most of the participants in the survey declared to administer a maximum of two adequate doses of BDZs. Most intensivists considered non‐iv BDZ administration only if vascular access was unavailable, reflecting a specialty‐related aptitude.

More impressive heterogeneity in treatment and ever‐decreasing adherence to the guidelines was reported in the literature as the treatment algorithm progressed (Nuñez et al. 2023). The collective results of the three fundamental trials on second‐line treatment (EcliPSE (Lyttle et al. 2019), ConSEPT (Dalziel et al. 2019), ESETT (Chamberlain et al. 2020)), demonstrating the non‐inferiority of LEV compared with PHT, have determined a change in current and preferred second‐line agents, and LEV has been added to treatment algorithms, including the recently revised Advanced Paediatric Life Support (APLS) guideline (Lyttle et al. 2019; Dalziel et al. 2019; Chamberlain et al. 2020; Bacon et al. 2023; Tyson et al. 2023). However, the management of second‐line therapy still requires careful interpretation and application of evidence (Appleton 2020).

In this survey, LEV was the preferred alternative to PHT as a second‐line treatment; moreover, 22 clinicians (6%), mainly pediatric emergency physicians from six different hospitals, indicated only LEV as a second‐line drug. Neurologists more frequently used LEV than intensivists, confirming the results of a recent survey focusing on the decision‐making processes during SE management in Turkey. There was no consensus among neurologists, intensive care, and emergency medicine specialists; familiarity with particular ASMs and etiologies was the most relevant factor influencing the attitudes (Dedeoglu et al. 2023).

MDZ can be used as a first‐, second‐, or third‐line drug based on the modality of administration and dosage. Concerning the widespread use in clinical practice, literature regarding recommended treatment pathways, dosing, and infusion rate is scarce (Francoeur et al. 2020).

While more recent studies focused on other possible routes of administration in first‐line therapy, several studies evaluated the efficacy of MDZ iv as a third‐line anesthetic drug for the treatment of RSE31; a gap exists in the evidence regarding its use and its efficacy in established SE. MDZ infusion could represent an interesting alternative to PHT and LEV due to its rapid onset of action; good cardiovascular stability; and transient, mild respiratory depression. It had a noteworthy impact on the timing of protective intubation. This aspect, among others, made prominent the role of the multidisciplinary team and the need to address the condition as a continuum.

In this survey, 38% of participants chose MDZ at a dosage < 0.23 mg/kg/h as a second‐line drug and 6.4% administered MDZ alone instead of another ASM at this step of treatment. This rate of choice may be positively influenced by the personal experience of some of the survey participants in the KETASER01 study, whose protocol contemplated the use of MDZ continuous infusion at low dosage among second‐line treatments before the randomization to a third‐line anesthetic (Rosati et al. 2016).

Continuous infusion of MDZ could represent an engaging alternative to PHT and even to LEV, considering its rapid onset of action, good cardiovascular stability, transient and mild respiratory depression, and low frequency of venous irritation.

Most participants declared using at least two ASMs before considering the third‐line treatment. Evidence justifying two second‐line ASMs, based on a single randomized controlled trial, was advised as an option by the new Advanced Paediatric Life Support (APLS) algorithm whenever the team was not ready to do an immediate anesthesia induction (Dalziel et al. 2019; Bacon et al. 2023). This recommendation highlighted the influence of organizational aspects on decision‐making and proved that more data are required regarding the timing of the transition to the third‐line treatment.

The survey participants’ choices regarding the statements “It is not my responsibility to decide” and “I do not manage…” expressed roles and specialty differentiation in the critical moment in which the “passing of the baton” occurred. The neurologists were primarily responsible for intercepting the transition, while the intensivists managed third‐line therapy.

In agreement with the literature, we found variability in the choice of anesthetics (Nuñez et al. 2023; Dedeoglu et al. 2023; Srivastava et al. 2023; Meyer et al. 2023). Our survey confirmed the increasing use of KE reported in the literature despite the scarcity of data from case reports and series, particularly in children (Gaspard et al. 2013; Keros et al. 2017; Jacobwitz et al. 2022).

KE was the third drug most often chosen by intensivists after propofol and MDZ, and it was the preferred anesthetic among pediatric neurologists. A recent study reported that KE was more frequently followed by seizure termination when administered as a first‐line anesthetic (61%) (Jacobwitz et al. 2022). This finding further underlined the particular relevance of the timing of the transition to third‐line treatment and the importance of sharing experience and knowledge in the multidisciplinary team.

Indeed, the aspects concerning multidisciplinary involvement, conceived as the results of the integrated combination of the care processes rather than the sum of the various parts provided by the various specialists, were considered a critical domain starting from the stage of the production of guidance documents in the SE approach (Cao et al. 2024).

It should be recognized that although treatment of SE often appeared sequential, it was actually a continuum of care with immediate cessation of seizure activity as the goal (Riviello et al. 2013).

Considering the clinical specificity of SE, specialists, including neurologists, critical care physicians, pediatricians, pharmacologists, were all involved in SE treatment and collaborated with specific roles related to their specialty at different times and faced with clinical pictures of different severity.

In real life, depending on resources and local organization, the presence of all specialties is habitually involved, and the assumption of roles in the multidisciplinary team can vary. Unique circumstances and scarcity of locally available resources may compromise the adherence to the sequential order and the correct timing of the traditional approach (first‐, second‐, and third‐line therapies); a particular etiology or severity of the clinical picture can explain the decision to change the sequence of treatment, moving directly to the third‐line treatment, when intensivists are involved and the context is safe; on the contrary, clinicians can be induced to postpone the progression to the anesthetic treatment, faced with the difficulty to correctly adequate the context and monitor the patient, due to the scarcity or unavailability of local resources.

Individual experience and skills related to specific specialties play an important role in the choices of treatment, emphasizing the necessity of a coordinated approach.

In the presented survey, in hospitals with an exclusively pediatric ICU, emergency pediatricians alerted neurologists and intensivists after the first‐line BDZs, and they were more prone to starting MDZ infusion at non‐anesthetic dosage, evidencing how the characteristics of the organizational system of the single hospital could influence the therapeutic work‐up of each stage of pediatric SE. The possibility of activating a multidisciplinary team, on duty or on call, was evaluated by questions on the availability of the medical personnel, EEG, and other diagnostic tools. Great answers variability revealed the gap in available personnel and diagnostic tool resources across Italy, difficulties in sharing knowledge, and the absence of standard and reliable procedures. Moreover, the variability in the number of participants in the survey from each hospital and the heterogeneity of the organizational system prevented us from stratifying data by level of intensity of care and correlating the organizational issues with therapeutic choices reliably.

Responses to the question “Do clinicians of your hospital use regional reference clinical pathways or guidelines for the diagnosis and pharmacological treatment of SE?” were often inconsistent among participants of the same hospital. Lack of awareness, understanding, or application was a cause of why practice does not keep pace with evidence‐based guidelines (Mayer et al. 2019).

Pediatric emergency physicians declared self‐confidence in managing patients with SE lower than child neurologists and intensivists. These results emphasized the importance of the involvement of all clinicians in processes aiming to improve quality. Timely feedback on information regarding the entire care pathway, from the first intervention to the outcome, in particular for RSE and SRSE, should be an integral part of all active stakeholders work.

This study has some limitations: the main one is common to all studies, survey‐based, and concerns the limited generalizability of the answers due to respondent selection issues and question‐related effects. Multiple‐choice questions concerning the choice of drugs did not require specifying the order of preference; therefore, it did not allow an analysis of the treatment pathway. The criterion of time in the therapeutic approach was not investigated. Time was one of the most important modifiable factors considered among studies that evaluated the improvement after protocols and guidelines dissemination. Since a positive relationship between seizure duration and delayed treatment has been demonstrated for pediatric and adult patients. The survey did not contain questions about years of participants’ experience, so it was impossible to analyze the influence of this factor on the treatment choice.

The participants did not represent the complete spectrum of specialists treating SE patient; however, we consider the response to the survey satisfactory to start the exploration of some issues about adherence to protocols and guidelines, focusing on the multidisciplinary perspective rather than the statistical representativeness of each single center and of the national territory.

We cannot exclude “desirable” answers, and we could not verify the accuracy of the answers. A lack of accuracy could be called into question to explain in particular the great inconsistency of the answers regarding the organizational aspects; lack of accuracy could be interpreted as an indication of the low importance, interest, and awareness in an aspect considered not directly dependent on one's specific responsibility.

Organizational aspects are fundamental in multidisciplinary approaches because they identify areas of variability depending on the availability of resources and therefore the applicability of guidelines. In particular, in the development of clinical pathways, awareness of organizational aspects should constitute a focus of educational training of all stakeholders of SE management.

How to best support the delivery of evidence‐based treatments in routine clinical practice is an ongoing concern not yet resolved; however, in addition to protocol‐guided therapies, the implementation of SE intervention teams is proposed as a strategy that may improve the timeliness of SE treatment (Stredny et al. 2018). The survey is the first investigation of pediatric SE management conducted in Italy. Good participation expresses the interest from all participating specialties, confirming the multidisciplinary approach. Active involvement of many different professional figures in sharing and discussing knowledge are fundamental clues to creating a consensus on the management of pediatric CSE, and it represents a mission for the IPSE group.

## Author Contributions

**Caterina Zanus**: conceptualization, methodology, data curation, investigation, supervision, writing–original draft, writing–review and editing. **Giulia Cannizzaro**: conceptualization, data curation, methodology, investigation, writing–review and editing. **Giacomo Danieli**: formal analysis, writing–original draft, writing–review and editing. **Angela Amigoni**: conceptualization, methodology, writing–review and editing. **Silvia Buratti**: conceptualization, methodology, writing–review and editing. **Francesca Izzo**: conceptualization, methodology, writing–review and editing. **Massimo Mastrangelo**: conceptualization, methodology, writing–review and editing. **Maria Cristina Mondardini**: conceptualization, methodology, writing–review and editing. **Paola Costa**: conceptualization, data curation, investigation, methodology, supervision, writing–original draft, writing–review and editing. **Anna Rosati**: conceptualization, investigation, writing–original draft, methodology, writing–review and editing, data curation, supervision. **Carla Marini**: conceptualization, investigation, methodology; writing–review and editing, data curation. **Lucia Fusco**: conceptualization, data curation, methodology, investigation, writing–review and editing.

## Ethics Statement

The study is based on an online survey and did not involve healthy people or patients. Thus, no ethical approval was required.

## Conflicts of Interest

The authors declare no conflicts of interest.

### Peer Review

The peer review history for this article is available at https://publons.com/publon/10.1002/brb3.70433

## Data Availability

The data that support the study findings are available from the corresponding author upon reasonable request.

## 1

Full list of participant hospitals:

1. IRCCS Azienda Ospedaliera Universitaria “A. Meyer,” Firenze
2. Città della salute Ospedale Infantile Regina Margherita (OIRM), Torino
3. IRCCS materno infantile Burlo Garofalo, Trieste
4. ASST Ospedale Papa Giovanni XXIII, Bergamo
5. ASST Fatebenefratelli Sacco Ospedale dei Bambini V. Buzzi, Milano
6. ASST Spedali Civili, Brescia
7. Ospedale Pediatrico G. Salesi Azienda Ospedaliero Universitaria Ospedali Riuniti, Ancona
8. Azienda Ospedaliera di Rilievo Nazionale di Alta Specializzazione (ARNAS) G.Brotzu, Cagliari
9. IRCCS Ospedale Pediatrico Bambino Gesù, Roma
10. IRCCS Università Cattolica del Sacro Cuore Fondazione Policlinico “A. Gemelli,” Roma
11. Azienda Universitario Ospedaliera. Policlinico Riuniti, Foggia
12. IRCCS—Ospedale Giannina Gaslini, Genova
13. IRCCS Azienda Ospedaliera Universitaria Policlinico Sant'Orsola Malpighi, Bologna
14. Ospedale Santa Chiara, Trento
15. Azienda Ospedaliera Universitaria Maggiore della Carità, Novara
16. ASST Settelaghi Ospedale F. Del Ponte, Varese
17. Azienda Ospedale‐Università, Padova
18. Azienda Ospedaliera Universitaria Integrata (Borgo Trento), Verona
19. Ospedale Provinciale di Bolzano, Bolzano
20. Presidio Ospedaliero ASST Rhodense, AO. G. Salvini Garbagnate Milanese, Rho
21. ASST Grande Ospedale Metropolitano Niguarda, Milano
22. IRCCS Policlinico di Milano, Ospedale Maggiore, Ospedale Ca' Granda, Milano
23. IRCCS Policlinico san Donato, san Donato Milanese
24. Ospedale Santa Maria degli Angeli, Pordenone
25. Azienda Ospedaliera di Rilievo Nazionale e di Alta Specializzazione (ARNAS), Di Cristina Ospedale dei Bambini, Palermo
26. Azienda Ospedaliera Universitaria, Parma
27. Ospedale Santo Bono, Napoli
28. Ospedale Pediatrico Giovanni XXIII, Bari
29. Azienda Ospedaliera Ospedale Santa Maria della Misericordia, Perugia
30. IRCCS San Gerardo dei Tintori, Monza
31. Azienda USL Val d'Aosta, Aosta
32. Ospedale Santissima Annunziata, Cosenza
33. Grande Ospedale Metropolitano, Reggio Calabria
34. Azienda Ospedaliera Universitaria Ospedale Civile SS. Annunziata, Sassari
